# Identifying the determinants of tourism receipts of Thailand and relevant determinant-determinant interactions

**DOI:** 10.1371/journal.pone.0308153

**Published:** 2024-08-01

**Authors:** Suree Khemthong, Pramote Luenam, Till D. Frank, Lily Ingsrisawang

**Affiliations:** 1 School of Management Science, Sukhothai Thammathirat Open University, Nonthaburi, Thailand; 2 Graduate School of Applied Statistics, National Institute of Development Administration, Bangkok, Thailand; 3 Department of Psychological Sciences, University of Connecticut, Storrs, Connecticut, United States of America; 4 Department of Statistics, Kasetsart University, Bangkok, Thailand; Middle Tennessee State University Jennings A Jones College of Business, UNITED STATES OF AMERICA

## Abstract

The study examined the determinants that affect tourism receipts in Thailand. To this end, quarterly data from eight main provinces of Thailand from the period 2015–2019 were used and constituted a repeated measures design. Accordingly, a generalized linear mixed model was applied for developing two different random intercept models by treating 1) province, and 2) a combination of province and calendar quarter as cluster-specific effects. It was found that determinants that increased tourism receipts were the number of visitors, the average cost per day, the length of stay of visitors, the presence of low-cost airlines, and a relatively low offence rate. Moreover, an increase in the number of visitors in the fourth quarter produced a higher amount of additional receipts as compared to a similar increase in the first quarter. Specifically, for Thailand attracting high-spending tourists and extending tourist visas for more than 30 days is recommended. Beyond Thailand, uncovering interaction effects as described above may help tourism agencies to focus their limited resources on the determinants that matter.

## Introduction

Over at least two decades, from 2000 to 2019, the importance of tourism increased worldwide in a more or less steady way when measured for example in terms of the worldwide income earned from tourism or the worldwide number of international travelers [1]. In 2019, about 10% of the global GDP came from the tourism and travel sector [2]. However, it is not just the substantial share that tourism has to the global GDP, it has also been suggested that tourism is a driving factor for economic growth [3]. By means of this mechanism the tourism industry can increase the GDP of a country [4–9]. In particular, it has been argued that tourism has a beneficial impact on economic development because it contains foreign currency [10]. Foreign currency, in turn, allows for the import of goods, which stimulates the overall economy [4]. These arguments have been developed prior to the COVID-19 pandemic that started at the beginning of the year 2020 and quickly spread out across the globe [11]. However, they are quite fundamental and are likely to hold for the post-COVID period as well. Therefore, irrespective of the COVID-19 pandemic and its impacts on tourism, it is important to study the factors that affect tourism for three main reasons. First, tourism is an important source of income. Consequently, it is crucial to understand the factors that determine this source of income. Second, a thriving tourism industry of a country is beneficial for the entire economy of the country. Therefore, it is worthwhile to study the conditions on which this driving factor depends upon. Third, to identify such determinants and to quantity their impacts is a necessary first step bevor they can be used in national plans and strategies to boost the tourism industry. In other words, governments and tourism authorities need evidence-based information in order to plan and promote tourism in their respective countries in a successful way. As a by-product, identifying the determinants that affect the revenues obtained from a particular tourism industry provides the industry with the tools to improve its performance since tourism industry performance, in general, is frequently measured in terms of the revenues that it generates.

The present study focuses on a key tourist destination that is outstanding both on a global and regional scale: Thailand. When taking international tourism revenue earned by a country as ranking criterion, Thailand was ranked worldwide among the top tourism earners on the fourth position in the year 2019 and was ranked first among all countries of the Southeast-Asian region [12]. Consequently, understanding the mechanisms underlying tourism demand in Thailand is not only important for the tourism industry, policy makers, and researchers within Thailand. Rather, the empirical question about what are the drivers of tourism demand of such a “tourism magnet” is a question of general interest. Traditionally, the Thai economy has heavily relied on the income from the tourism sector. During the period 2009 to 2019, just as the worldwide number of international travelers increased, the number of foreign tourists, who visited Thailand, increased from 14.1 million in 2009 to 29.9, 32.6, 35.4, 38.2, and 39.9 million in 2015 to 2019, respectively [13]. As a result, the tourism receipts raised up from 0.51 trillion Thai baht in 2009 to 1.45, 1.64, 1.82, 1.78, and 1.91 trillion Thai baht in 2015 to 2019, respectively [13]. These receipts contributed to Thailand’s GDP ranging from 9% to 18% [14]. The 18% value is way above the 10% worldwide value of 2019 mentioned above and demonstrates the importance of the tourism sector for Thailand. During the COVID-19 pandemic period of 2020 to 2022, the number of foreign tourists, who visited Thailand, decreased to only 6.7, 0.4, and 11.2 million, respectively, [15]. Likewise, the contribution of the tourism sector to Thailand’s GDP was at relatively low levels of 5.65%, 2.02% [16] and 5.66% [17]. As such, as of October 1, 2022, Thailand is officially fully reopened for tourists [18] and the Thai tourism industry is currently witnessing a rapid recovery. In the first half of the year 2023, Thailand welcomed about 13 million visitors, which is more than the total of 11.2 million of the entire previous year [19]. At the end of the year 2023 Thailand had welcomed at total of 28 million visitors in total [19]. On the one hand, this number was still 30% below the 2019 pre-COVID number. On the other hand, it means that the tourist numbers more than doubled within only one year, which can be seen as a clear sign of recovery. In view of the re-established convenient conditions for tourists to enter Thailand and the re-discovered interest of tourists in Thailand, one of the strategic objectives of TAT’s most recent strategic plan is to increase the volume of tourists’ expenditure [20].

In order to support such policy and market plans, the current study aimed to identify the determinants of tourism receipts in Thailand. In this regard, a study is missing that examined the period during which tourism worldwide and in Thailand was at its peak value just before the 2020 pandemic. Similarly, studies on tourism in Thailand that attempted to scan a comprehensive, multivariable space are rare. In particular, a number of studies have primarily focused on factors like GDP and exchange rate and considered relatively small sets of possible factors. Moreover, interaction effects have been frequently neglected. That is, research on tourism demand for Thailand just as for other countries across the globe has typically focused on the impacts of determinants in isolation and neglected possible interactions between determinants. In this context, a generalized linear mixed modeling (GLMM) approach to identify not only interactions but also the presence of unknown factors and to determine the possibility of a fine-grained characteristics underlying tourism demand has not yet been applied to data from Thailand. In general, in the field of tourism research the power of this kind of modeling analysis has not been fully appreciated (for an exception, see, e.g., [21]). Previous studies on tourism demand in Thailand have primarily focused on Thailand as a whole. Consequently, while they have yielded important findings, they could not be specific as to which regions or provinces within Thailand their findings actually applied. Traditionally, the main provinces that attract tourists in Thailand are Bangkok, Phuket, Chonburi, Surat Thani, Chiang Mai, Songkhla, Krabi, Ayutthaya, Phangnga, Prachuap Khiri Khan, and Samut Prakan [22]. In order to give the analysis of tourism demand more specificity, the current study focused on a number of provinces rather than on Thailand as a whole. More precisely, the focus was on eight of those main provinces for which air pollution data in terms of particulate matter levels were available: Bangkok, Phuket, Chonburi, Surat Thani, Chiang Mai, Songkhla, Phra Nakhon Si Ayutthaya, and Samut Prakan. In addition, in contrast to previous studies on determinants of tourism demand in Thailand, the aim of the current study was to identify those determinants for a period just before the pandemic took place, which is the period that most likely is most relevant for the ongoing post-COVID recovery period in Thailand and is the period during which Thai tourism was at its peak (see above). While previous periods have been researched (see the literature section below), this important period has not yet been analyzed in detail. In order to conduct such an analysis, the objective was to take a relatively broad repertoire of promising candidate determinants into account that have not been looked at in combination in previous studies.

In the current study, we selected variables that have been found in previous international studies to be useful determinants of tourism demand. We hypothesized that most of them would turn out to have statistically significant effects. However, we also hypothesized that tourism in Thailand should have its own signature such that a few candidate determinants should turn out to be irrelevant. Further objectives of the current study were to quantify and discuss impacts of relevant determinants, to identify possible interaction effects between determinants, identify the presence of unknown factors, and examine the possibility of a fine-grained characteristics underlying tourism demand using an appropriate dual-model GLMM approach that involved a baseline and a fine-grained model. The methodological GLMM approach had also the potential to shed light on tourism research beyond Thailand. That is, the aim was to illustrate for the case study on Thailand how determinant-determinant interactions and other aspects of generalized linear mixed models can be identified, interpreted and used for establishing recommendations.

In summary, the objectives of the present study were the following. We aimed to identify the determinants of tourism demand in Thailand as measured in terms of tourism receipts with the help of two appropriately defined models that both came with a well-defined, specific study area and examined the period just before the pandemic but differed in detail. We also aimed to identify explicitly given factors, their quantitative impacts and their interactions and to uncover the presence of unknown factors. In doing so, our goal was to establish an anchor point that can be used in the literature to fill a gap in this regard that can be used, on the one hand, by Thai tourist authorities for policy making and, on the other hand, by researchers in the field of tourism demand as a blueprint for future studies.

The remainder of this paper is organized as follows. In the second section a literature review is presented that also motivates the variables that were used in the current study and presents relevant literature specific for tourism in Thailand. The third section presents the data and the methodology used in the current study. The results are presented and discussed in the fourth section. Conclusions with an eye on policy implications are given in the final, fifth section.

## Related literature

### Determinants of tourism demand and receipts

Tourism demand can be measured by means of several variables such as tourism receipts, the number of tourists (or arrivals), and the number of employees in the tourist industry [23]. This section reviews some determinants of tourism demand used in the literature. The utilization of those variables in the literature motivated us to use those variables in the current study. The variables are listed in Table 1 together with the relevant references. While the current study focuses specifically on tourism receipts, the determinants of tourism demand in general can be considered as promising candidate variables that affect tourism receipts.

**Table 1:** Factors used in the current and previous studies.

Factors	Refs
Foreign visitors Tourist cost of living measure: average cost per day Calendar season Calendar quarter Occupancy rate Length of stay Number of tourist attractions Having low-cost airline Exchange rate Air pollution and pollution in general Crime rate Size of tourist destination Tourism confidence index	[21, 24, 25] [26, 27] [28–31] [32, 33] [25] [29, 34–40] [24, 41] [42, 43] [44, 45] [31, 46] [47, 48] [49] [50]

Two obvious determinants are the number of foreign visitors per time period of consideration and the average cost per day. Nursini et al. [24] investigated among other things the effect of the number of tourists on the tourism sector revenues in the Gowa district of Indonesia under pre-COVID circumstances. They found that the number of tourists had a significant and positive effect on tourism revenue. Likewise, Raharti et al. [25] showed for data from Yogyakarta city, Indonesia, for the period of 2005 to 2018 that the number of tourists had a significant effect on the revenues coming from the tourism sector. More recently, Medai et al. [21] showed that roughly speaking the number of visitors had a positive effect on the tourism revenues obtain from hot spring areas of Japan when focusing on the year 2018 and considering the major ten Japanese hot springs. When studying factors determining the number of foreign visitors, it has been suggested to take a measure for the cost of living for tourists at their destinations into account. If such as measure is not available, the consumer price index of the destination country may be used as a proxy [26]. In fact, the latter factor has been used in several studies on tourism demand [27] but has the disadvantage that it is typically defined on the country level and not available for local regions of a country of interest. In the current study, a locally defined measure similar to the cost of living factor was used: the average cost per day. The factor average cost per day measures the daily expenditures that tourists make during their visits in a country. Therefore, the hypothesis is motivated that there is a positive association between the amount of daily spending observed in a certain region and the corresponding observed tourism receipts. Another obvious variable is tourism season [28–30]. For example, the study by Zhang et al. [31] nicely demonstrates that for tourism in Beijing, China, during the high season period from April to October tourist arrivals are relatively high as compared the arrivals during the low season from November to March. Calendar quarter with four quarters in the year is a more fine-grained measure than the tourism season variable. In addition, the measure is not biased by the specifics of a destination country of interest. The four quarters of the year are defined in the same way across all countries. It is a frequently used factor in statistical economic models [32]. It is a special case of the factor time-period that again is a classical factor considered in statistical modeling of economic data [33]. Therefore, calendar quarter is a promising candidate determinant of tourism demand.

The aforementioned study by Raharti et al. [25] also examined the impact of the hotel occupancy rate on tourism revenues. Just as for the number of tourists, it was found that the hotel occupancy rate had a significant, positive effect on revenues. The length of stay is a variable that has been frequently studied [29, 34–40]. However, the effect of the variable seems to depend on the precise circumstances. For example, Gomez-Denis et al. [34] found that tourists on the Canary Island during the years 2009 to 2012 who stayed for longer periods also reported higher spendings. In contrast, in the study by Massidda et al. [37] on British travelers the opposite effect was found. Length of stay had a negative effect on expenditure. Likewise, in the study by Mehmetoglu [51] it had a negative impact on tourists’ expenditure.

Furthermore, there are factors that make provinces more attractive such as the number of tourist attractions, the presence of airports, and the availability of low-cost airlines. These factors serve as promising candidates for determinants of tourism receipts. For example, Nursini et al. [24] found that the number of attractions had a significant effect on tourism revenue. Similarly, Zhang et al. [41] classified cities into different categories according to the number of attractions and found that tourist behavior was different for different classes. Bieger and Wittmer [52] argued that airports indicate to tourists possible tourist destinations. Consequently, provinces that have an airport may benefit from higher tourism revenues as compared to comparable regions without airports. Not only having an airport, but also having a low-cost airline at that airport may increase the attractivity of a destination. In fact, Gomez-Deniz and Perez–Rodriguez [42] showed for travelers from the United Kingdom to the Canary Islands that the factor low-cost airline had a significant effect on the expenditure of the travels. It decreased (rather than increased) expenditure, which makes sense when the effect is seen from the perspective of the traveler. In a more general sense, Eugenio-Martin and Inchausti-Sintes [43] argued that low-cost travel options should affect the spending of tourists at their destinations because when the budget of travelers is fixed then paying less for travel allows travelers to spend more on other items at the destination. They found some evidence for their hypothesis when analyzing data, again, from travelers to the Canary Islands. Appropriately defined savings-transfer ratios were found to range between 10.3% and 46.1%.

A factor that makes a country as a whole more attractive as tourist destination is the exchange rate between the currency of that destination country and currency of the country of the tourist. For example, Barman and Nath [44] found that tourist arrivals in India increased during the years from 2001 to 2015 when the exchange rate against the Indian Rupee increased in favor of the tourists. Lee and Hassan [45] found for the period of 2003 to 2017 a causal link between tourist receipts from Malaysia and the exchange rate between the Malaysian’s Ringgit and currencies of the visitors’ countries considered in their study.

Two factors that make tourist destinations less attractive are air pollution and a high crime rate. For example, in the aforementioned study on tourism in Beijing, China, Zhang et al. [31] also showed that tourist arrivals were relatively low during periods of haze pollution. More precisely, they found that the number of tourist arrivals was inversely proportional to the concentration of particulate matter. This effect is not surprising because there is a continuously growing literature on the negative health effects of particulate matter (see, e.g., Varapongpisan et al. [53], and references therein) such that the general public becomes more and more aware of the negative effects of air pollution. Not only air pollution but all forms of pollutions at a certain destination are likely to scare tourists away from that destination. For example, Jang et al. [46] studied the impact of the July 2011 marine debris pollution event on the tourism revenue in Geoje Island, South Korea. They found that the number of visitors decreased from about 890,000 persons in the year 2010 to 560,000 persons in the year 2011 when the pollution took place. That is, the number of visitors dropped to a level of 63% of the non-pollution period. Just as pollution has a negative impact on tourism, tourists are likely to avoid destinations with a relatively high crime rate or offence rate. In general, the destination image that may be affected by safety concerns plays an important role in tourism research [47, 48].

The size of a tourist destination region may be considered as another possible determinant for tourist demand, in general, and tourist receipts, in particular. In this context, Khusnutdinova et al. [49] noted that cities offer places that can be visited by tourists, events that can be attended by tourists, and other opportunities that can be taken by tourists in any weather. That is, irrespective of season and weather larger cities and related to that larger destination regions may have more to offer to tourists and, consequently, may attract more tourists as compared to smaller cities and regions. Finally, previous research has examined the role of the tourism confidence index (TCI) on tourism demand. For example, Croce [50] showed that international tourist arrivals could be better predicted when TCI was taken into account.

The impact of the COVID-19 pandemic on domestic and international tourism has been extensively studied [54–58]. There is a general agreement that the outbreak of the pandemic had psychological effects on tourists worldwide [54]. A specific effect of the pandemic that has been frequently reported is an increase in risk perception such that tourists are less willing to travel for fear of a COVID-19 infection [54, 55, 58]. For example, in the study by Li et al. [58], domestic tourism demand in China during the year 2020 was negatively associated with the number of confirmed COVID-19 cases at the tourist destinations when seen relative to the cases of the tourists’ hometowns. This more recent body of literature on COVID-19 related travel risks may be seen in the context of the more traditional studies that examined the role of safety concerns for the decision making of tourists (see above). However, while it is plausible to assume that general safety concerns will be an all-time factor that affects tourists’ decisions, this is not so obvious for the fear for COVID-19 infections.

In the aforementioned studies various methods have been used. Regression models have been frequently used to identify relevant factors affecting tourism demand [24–26, 28–30, 36, 37, 40, 43, 44]. They have been also used as departure points for building more sophisticated models [34, 38, 42, 45]. For example, they have been applied to determine Granger causality among the factors being considered [45] or to determine the distribution of the variable measuring tourism demand [34, 42]. In this context, economic studies involving regression models have often focused on particular economic perspectives and, in doing so, have used models exhibiting relatively small numbers of factors (e.g., between 3 and 5) [24, 25, 44, 45]. Structural equation modeling has been used in the field of tourism demand research as an alternative way to address the aforementioned issue of causality [48]. In order to study determinants of tourism demand, time series models have been utilized as well [27, 50]. Finally, a few studies have used generalized linear mixed models and panel analysis models to identify those tourism demand determinants [21, 41]. Overall, the methods that have been applied are quite diverse. The methods typically have been selected such that they meet the needs and goals of the studies at hand.

### Tourism research from Thailand

As mentioned in the introduction, several studies have shown that tourism has been a driving factor for economic growth in general. Specifically for Thailand, Untong [59] and Jiranyakul [60] showed that during selected periods of 1980–2012 [59] and 2016–2017 [60] there was a causal relationship between economic growth and the growth of the tourism industry when using appropriate variables to describe both sectors. Most recently, Phadkantha et al. [61] confirmed the aforementioned findings by showing again that tourism is a driving factor of Thai economic growth.

As far as determinants of tourism demand in Thailand are concerned, Zhang et al. [62] examined factors that affected tourist arrivals in Thailand during the 20 years period from 1987 to 2006. As part of their comprehensive study, they found that the exchange rate (Thai Baht to US Dollars), the financial crisis during the years 1997 and 1998 and the SARS outbreak in 2003 affected the tourist arrivals. Accordingly, when the exchange rate was in favor of the foreign tourists then arrivals were relatively high. Furthermore, the financial crisis as well as the SARS outbreak negatively impacted the number of arrivals. Thailand is famous for beaches and islands. Not surprisingly, several studies have focused on understanding the dynamics of tourism on Thai islands. Park et al. [63] studied tourism determinants for Phuket and used a questionnaire that was handed out to tourists visiting Phuket in 2007. Participants were asked to rate several motivational items (i.e., travel reasons) to travel to Phuket on five-point Likert scales. Among the many statistically significant items that motivated tourist to visit Phuket were the safety and security of the facilities that they visited, the clean and clear sea and the good air quality, and the pricing of travel packages including airfare, food, and accommodation. These findings echo what has been stated in the previous section that air pollution, crime rate, and the availability of relative cheap travel options are promising determinants of tourism demand. Likewise, Dodds et al. [64] conducted a survey on tourists of the Thai island Koh Phi Phi to determine the factors that are most relevant for them to travel to the island. They found that cleanness of the beaches, cleanness of general destination, safety, value for money, and the cheapness of the destination were among the first 10 most relevant items. Returning to Thailand as a whole as destination country, Harasarn and Chancharat [65] showed that safety concerns have an impact on international tourist arrivals in Thailand. They used annual data from 1981 to 2014 and showed that tourist arrivals were negatively impacted by periods of political unrest in Thailand. Chaivichayachat [66] identified further factors affecting tourism demand in Thailand. To this end, data from 2009 to 2016 of foreign tourist arrivals were used. Exchange rate, crime rate, and the calendar season were among the factors that had a significant effect on the number of tourist arrivals. In a more recent study, Chulaphan and Barahona [67] examined determinants for tourism demand in Thailand using expenditure data of tourists visiting Thailand during 2010 to 2017. As part of their comprehensive study, they found that the price difference between the tourist’s country and Thailand positively affected expenditure. This finding is consistent with the aforementioned studies showing that the exchange rate between a tourist’s country and the destination country may affect how much a tourist is spending. Importantly, Chulaphan and Barahona [67] investigated the effect of corruption in Thailand as seen from the perspective of the tourist. Not surprisingly, they found for the time period under consideration that when corruption in Thailand was perceived as high then expenditure was reduced. This effect is consistent with the statement made above that a high crime rate in a destination country is likely to constitute a negative factor for tourism. Finally, the role of tourist attractions and how convenient they can be reached was explored in a study conducted during the COVID-19 period of the year 2022. Srisawat et al. [68] interviewed foreign tourists visiting Thailand in 2022 by means of questionaries. As part of their comprehensive study, they found that the factors attraction and accessibility influenced travel behavior of those tourists. This finding supports to some extent the findings reviewed in the previous section that the number of tourist attractions, on the one hand, and the presences of low-cost airlines, on the other hand, positively affect tourism.

## Data and methods

### Data

The data in this study was collected for the five-year period of 2015 to 2019. Quarterly data was used. Data was collected for the eight main provinces mentioned in the introduction: Bangkok, Phuket, Chonburi, Chiang Mai, Phra Nakhon Si Ayutthaya, Samut Prakan, Songkhla, and Surat Thani. Tourism receipts (in million baht) as outcome variable was taken from the database of the Ministry of Tourism and Sports of Thailand [69]. The factors reviewed in the previous section and listed in Table 1 were used as potential independent variables and were collected as follows. Data of average length of stay (in days), hotel occupancy rates (%), average cost per day (in Thai baht) and the number of foreign visitors (in persons) were extracted again from the databases of the Thai ministry of Tourism and Sports [69]. Province size data (small, middle, large) was collected from the website of the Ministry of Interior of Thailand [70]. Thailand tourism confidence index values were taken from the website of the Tourism Council of Thailand [71] and were converted into binary data (greater or equal 100 vs. less than 100). The number of attractive locations in each province was determined with the help of the website of Traveloka [72]. The information whether provinces had low-cost airlines (yes/no) was obtained from the website of the Travel Company Mundo Nomada [73]. The exchange rate from US dollar to Thai baht, the offence rate of life per 100,000 people, and the level of particulate matter up to 10 micrometers in size (PM_10_) were taken from the databases of the Bank of Thailand [74], Royal Thai Police [75], and the Pollution Control Department of Thailand [76], respectively. PM_10_ values were converted into two levels (PM_10_ ≤ 50 μg/ m^3^ and PM_10_ > 50 μg/ m^3^). High season was defined as the period of the first and fourth calendar quarters (during which international tourists mostly visit Thailand) [77]. Accordingly, low season was defined as the period of the second and third quarters of the year. For all variables the sources and data can be found in the S1 File and S1 Data. The data set contained for every province 20 measurements across quarter and year. Table 2 provides descriptive statistics of the continuous variables used in the study. Table 3 provides characteristics of the provinces considered in this study. More detailed descriptions of the study areas can be found in the S1 File.

**Table 2:** Continuous variables used in the present study.

Variables	Mean (SD)
Receipts (in million baht)	38960 (50851)
Foreign visitors (in persons)	1686291 (1721170)
Average cost per day (in baht)	4216 (1821)
Offence rate	7.7 (1.0)
Average length of stay (days)	3.7 (1.2)
PM_10_ (μg/ m^3^)	40.25 (14.21)
Exchange rate	33.37 (1.71)
Number of tourist attractions	22.5 (7.9)
Occupancy rate	71 (9)
Thailand tourism confidence index	97.8 (3.5)

**Table 3:** Provinces and some of their variables considered in the present study.

Province	Region of Thailand	Size of province	Having low-cost airline	Average cost per day (in baht)	Foreign visitors (in persons)	Receipts (in million baht)
Bangkok	Central	Large	Yes	5611 (673)	5643081 (718456)	142180 (29284)
Samut Prakan	Central	Small	No	1791 (137)	280872 (156118)	721 (295)
Phra Nakhon Si Ayutthaya	Central	Medium	No	2061 (358)	488261 (38856)	1366 (325)
Chonburi	East	Medium	Yes	4880 (780)	2225977 (348572)	44598 (16301)
Chiang Mai	North	Large	Yes	4161 (353)	780904 (112349)	9274 (1763)
Surat Thani	South	Large	Yes	3785 (477)	871380 (122275)	17799 (4079)
Phuket	South	Small	Yes	7230 (1626)	2512547 (620489)	88370 (34635)
Songkhla	South	Large	Yes	4209 (438)	687303 (82923)	7369 (1382)

For continuous variables mean and SD are shown.

## Methods

Tourism receipts were considered as dependent variable. An explorative analysis on the entire data set was conducted to address the distribution of the receipts, variable selection and multicollinearity issues. To this end, the tourism-receipts histogram was computed. In view of previous studies, a right-skewed distribution was expected. Following de Jong and Heller [33], a Gamma distribution was used to model the receipt data in case that the initial hypothesis could be confirmed. The model construction involved an initial and final pool of independent variables. The initial pool of variables considered for modelling was given by the 13 variables described in the previous section: number of foreign visitors (in one-hundred-thousand person), average cost per day (in 1,000 Thai Baht), calendar season and calendar quarter, hotel occupancy rate (in %), length of stay (in days), number of attractive locations, presence of low-cost airlines, exchange rate, PM_10_ levels (categorical), offence rate (in incidents per 100,000 people), size of tourist destination (categorical), and tourism confidence index (categorical). To check for variable importance, those variables were subjected to a procedure as described in Hosmer and Lemeshow [78]. Accordingly, a univariate analysis was conducted to model the effect of each independent variable on the receipts outcome. Independent variables that showed a statistical significance at a 0.25 level were preliminarily accepted as potential predictors. All other variables were rejected. To test for multicollinearity, for all preliminarily accepted variables the variance inflation factor was computed. Only variables with factors less than 10 were accepted. In doing so, a final pool of variables was obtained (see Results section) that were included in the GLMM models. Two GLMM models were considered. The baseline GLMM model involved a log link function (see also [21]) and was given by:

$$\ln\;E({\mathrm{receipts}}_{pt}|u_{p})=\beta^{\left(0\right)}+\sum_{k=1}^{m}\beta^{\left(k\right)}x_{pt}^{\left(k\right)}+\sum_{k,l=1;k\neq l}^{m}\beta^{\left(kl\right)}(x_{pt}^{\left(k\right)}\times x_{pt}^{\left(l\right)})+u_{p}$$ (1)

In Eq (1) the variable receipts_*pt*_ describes the receipts of province *p* at time *t*, where *t* measures time in calendar quarters, *β*^(0)^ denotes the fixed intercept, indices *k* and *l* count the *m* independent variables of the final pool, *x*^*(k)*^_*pt*_ with *k* = 1,…,*m* denote the independent variables of that pool measured for province *p* at time *t*. Moreover, *β*^(*k*)^ and *β*^(*kl*)^ denote the regression coefficients of the main and interaction effects of factor *k* and factors *kl*, respectively. Finally, according to the general random-effect modeling approach of longitudinal data [79], *u*_*p*_ denotes the province-specific random effect and is assumed to be normally distributed with zero mean. In order to tests the possibility that for different provinces different quarters play important roles, a more fine-grained model was constructed as recommended in Ref. [80] and the baseline model (1) was extended as follows:

$$\ln\;E({\mathrm{receipts}}_{pqt}|u_{pq})=\beta^{\left(0\right)}+\sum_{k=1}^{m}\beta^{\left(k\right)}x_{pqt}^{\left(k\right)}+\sum_{k,l=1;k\neq l}^{m}\beta^{\left(kl\right)}(x_{pqt}^{\left(k\right)}\times x_{pqt}^{\left(l\right)})+u_{pq}$$ (2)

In the fine-grained model defined by Eq (2) receipts_*pqt*_ describes the receipts of province *p* for quarter *q* during the year *t*. Just as for the baseline model, *β*^(0)^ in Eq (2) denotes the fixed intercept, indices *k* and *l* count the *m* independent variables of the final pool and *x*^*(k)*^_*pqt*_ denotes the *k*th independent variable measured for province *p*, quarter *q*, and year *t*. Accordingly, *β*^(*k*)^ and *β*^(*kl*)^ denote again the regression coefficients of the main and interaction effects of factor *k* and factors *kl*, respectively. Likewise, *u*_*pq*_ denotes the random effect specific for province *p* and quarter *q*, which is assumed to satisfy a normal distribution with zero mean. As indicated in Eqs (1) and (2), the independent variables were entered into the models on linear scales such that the exponentiated coefficients exp(*β*^*(k)*^) of the main effects quantify how much receipts increase (*β*^*(k)*^>0) or decrease (*β*^*(k)*^<0) for one-unit increases of the corresponding independent variables. Furthermore, in the context of the GLMM approach, the aforementioned Gamma distribution *G* is parameterized such that receipts are distributed like *G* with mean values defined by the mean values occurring in the left-hand-side of Eqs (1) and (2). In order to compare the performance of the two models two measures were determined: the loglikelihood and the Akaike information criterion (AIC) measure. The latter measure is considered to be the more conservative one.

## Results and discussion

As expected, the receipt data followed a right-skewed distribution (see the S1 File). The univariate analysis accepted all variables except for size of provinces (see the S1 File). All 12 remaining variables exhibited variable inflation factors less then 10 (see the S1 File). Accordingly, those variables did not exhibit any severe degree of multicollinearity. Consequently, the GLMM models defined by Eqs (1) and (2) included the following 12 factors: number of foreign visitors, average cost per day, calendar season and calendar quarter, hotel occupancy rate, length of stay, number of attractive locations, presence of low-cost airlines, exchange rate, PM_10_ levels, offence rate, and tourism confidence index.

The estimation results for models (1) and (2) are shown in Tables 4 and 5. Table 4 summarizes model estimates for all statistically significant main and interaction effects. Overall, the two models showed consistent effects in the sense that the statistically significant effects did not differ across the baseline and the fine-grained models except for a few exceptions (see below). The RMSE of model 2 was lower than the RMSE of model 1, indicting that the fine-grained model 2 fitted the data better. Likewise, the loglikelihood value of model 2 was higher as compared to model 1, indicating again a better fit of model 2. Importantly, the value of the Akaike information criterion was lower for model 2 indicating that the latter model produced a better fit even when taking a more conservative point of view. The fact that model 2 fitted the data better than model 1 suggests that the random effects on the level of provinces of the baseline model 1 cannot capture all the subtle characteristics of the receipts data under consideration. In contrast, the random effects specific to both provinces and quarters of the fine-grained model 2 seem to be able to capture more of those characteristics, which then leads to a lower RMSE, a higher loglikelihood value, and a lower AIC score.

**Table 4:** Estimation results for the baseline and fine-grained GLMM models 1 and 2.

	Model 1		Model 2	
	Estimated *β* (SE)	e^*β*^	Estimated *β* (SE)	e^*β*^
Foreign visitors	0.0464** (0.0041)	1.048	0.0312** (0.0048)	1.032
Average cost per day	0.6426** (0.1100)	1.901	0.3886** (0.0858)	1.475
Average length of stay	0.1771** (0.0310)	1.194	0.1544** (0.0247)	1.167
Having low-cost airline	1.5377** (0.2509)	4.654	1.9987** (0.1841)	7.379
Offence rate	- 0.0472** (0.0121)	0.954	- 0.0599** (0.0067)	0.942
Occupancy rate	0.0250** (0.0054)	1.025	0.0283** (0.0039)	1.029
Calendar Quarter				
Q1 (reference group)	0	1	0	1
Q2	-0.1399** (0.0442)	0.869	-0.5040 (0.2238)	0.947
Q3	-0.1272* (0.0495)	0.881	0.0875 (0.2245)	1.091
Q4	-0.2331** (0.0458)	0.792	-0.3181 (0.2317)	0.728
Foreign visitors × quarter				
• Foreign visitors × Q1 (ref. group)	0	1	0	1
• Foreign visitors × Q2	0.0048** (0.0017)	1.005	0.0027 (0.0056)	1.003
• Foreign visitors × Q3	0.0015 (0.0017)	1.001	-0.0082 (0.0058)	0.992
• Foreign visitors × Q4	0.0137** (0.0021)	1.014	0.0225** (0.0067)	1.023
Average cost per day × occupancy rate	- 0.0062** (0.0013)	0.994	-0.0039** (0.0011)	0.996
Obs	160		160	
RMSE	0.125		0.054	
Loglikelihood	-1422		-1368	
AIC	2876		2768	

Note

* p<0.05

** p<0.01. Standard errors were calculated from variance-covariance matrices.

**Table 5:** Estimated fixed and specific random intercepts for models 1 and 2.

Model 1		Bangkok	Chiang Mai	Chon Buri	Ayutthaya	Phuket	Samut Prakan	Songkha	Surat Thani
	*β* ^(0)^	*u*_1_	*u*_2_	*u*_3_	*u*_4_	*u*_5_	*u*_6_	*u*_7_	*u*_8_
	4.6800** (0.4802)	-0.3892* (0.1805)	-0.1483 (0.1310)	0.3554** (0.1163)	0.2149 (0.1967)	0.3504** (0.1236)	-0.2157 (0.1967)	-0.2781* (0.1353)	0.1074 (0.1311)
Model 2									
	*β* ^(0)^	*u*_1q_	*u*_2q_	*u*_3q_	*u*_4q_	*u*_5q_	*u*_6q_	*u*_7q_	*u*_8q_
	4.837** (0.3604)								
*q* = 1		0.0904 (0.2396)	-0.4553** (0.1633)	0.3827* (0.1503)	-0.0945 (0.1947)	0.6989** (0.1640)	0.0791 (0.1941)	-0.6289** (0.1642)	-0.0752 (0.1613)
*q* = 2		0.1825 (0.2343)	-0.4485** (0.1610)	0.4010** (0.1517)	0.5006* (0.1943)	0.5952** (0.1542)	-0.4980* (0.1958)	-0.5201** (0.1616)	-0.2156 (0.1654)
*q* = 3		0.6367* (0.2523)	-0.3747* (0.1590)	0.2105 (0.1511)	0.1740 (0.1939)	0.4685** (0.1534)	-0.3670 (0.1956)	-0.5632** (0.1635)	-0.1877 (0.1591)
*q* = 4		-0.2098 (0.2373)	-0.4070** (0.1653)	0.2105 (0.1549)	0.4771* (0.1948)	0.4587** (0.1948)	-0.2742 (0.2010)	-0.3935* (0.1710)	0.1353 (0.1602)

Note

** p < 0.01

* p < 0.05

For the baseline as well as for the fine-grained model, the number of foreign visitors was positively associated with tourism receipts (see Table 4). This observation is consistent with previous studies from Indonesia [24, 25] and expected since any tourist that visits a destination country spends at least some amount of money at the destination and, consequently, increases the income of that country due to tourism. Therefore, the question is not whether income from tourism increases with the number of tourists. Rather, the first question is whether such a relationship is statistically significant, which means, whether it makes a key contribution that can be identified against the randomness naturally involved in all kind of data. If so, the second question is what quantitative insights can be gained from that observation. As far as the first question is concerned, our dual-modelling study produced significant effects across both models, confirming that the observed effect of the number of tourists was not a by-chance effect. From the exponentiated factors in Table 4, it can be learned that according to Eqs (1) and (2) every one-hundred-thousand increase in foreign visitors increases tourism receipts by 3.2% - 4.8%. In this context, the baseline model 1 predicts an increase of 4.8% that is by a factor 1.5 higher as the 3.2% increase predicted by the fine-grained model 2. This observation suggests that neglecting to address properly the supposed fine-grained structure underlying the tourism receipts under consideration leads to a slight overestimation of the effect of the number of tourists.

Qualitatively, both models showed that visits with higher average cost per day made a higher contribution to tourism receipts than visits with lower average cost per day. While this effect was expected, our analysis showed that quantitatively different estimates can be obtained when different modelling strategy are used. More precisely, the regression coefficient *β* of the baseline model was by a factor 1.7 higher than the coefficient of the fine-grained model (see Table 4). This implies that the baseline model 1 predicts that for every 100 Baht increase receipts increase by 6.6%, while the fine-grained model 2 predicts only an increase by 4.0%. Again, just as for the effect of the number of tourists, the fine-grained model produced a more conservative estimate. Irrespective of this difference, the general question arises what are the characteristics of tourists who tend to spend more as compared to budget tourists who tend to spend less? For example, Jang et al. [30] showed that among Japanese tourists who travelled abroad in 1995 big spenders were honeymooners and people combining business trips with pleasure trips. Moreover, the study showed that spending increased with age (see also Mehmetoglu [51], Jang et al. [36]). While these findings do not necessarily generalize to visitors coming to Thailand, the study by Jang et al. [30] provides a hint what kind of tourist could be willing to spend more.

From the regression coefficients of the factor length-of-stay reported in Table 4 and Eqs (1) and (2) it follows that tourist who stayed longer in Thailand increased Thai tourism receipts. The regression coefficients of both models were positive and differed by a relatively small amount (a factor of 1.2). According to Eqs (1) and (2), making tourist to stay 1 day longer, increases tourism receipts between 17% (model 2) and 19% (model 1). Again, the fine-grained model (model 2) produced a less dramatic, more conservative estimate as compared to the baseline model (model 1). As mentioned in the Related Literature Section, the length of stay is a variable that has produced seemingly conflicting results in the previous literature. For example, Gomez-Denis et al. [34] and Jang et al. [36] found a positive effect of length of stay on spending, consistent with our finding. In contrast, Mehmetoglu [51] and Massidda et al. [37] found that length of stay had a negative impact on expenditure. In order to explain such differences, first of all, the dependent variable should be revisited used by the different research groups. In the current study as well as in Gomez-Deniz et al. [34] and Jang et al. [36] total or cumulative money values were considered, namely, the receipts received during a quarter (current study) and total trip expenditure [34, 36, 37], respectively. In contrast, Massidda et al. [37] used as measure for tourism demand the expenditure per day. In general, when the length of stay increases and results in a decrease of the expenditure per day, then this does not necessarily imply that the total expenditure per trip decreases. It might do so. It might also increase. The same argument applies to the study by Mehmetoglu [51], which used trip expenditure per day as measure of interest. In short, our finding of a positive effect of length of stay on quarterly observed receipts is not necessarily in contradiction with studies that have found a negative effect of length of stay on daily measures of tourist expenditures.

According to our analysis, having a low-cost airline and a relatively low offence rate were also factors that were beneficial to increase receipts (see Table 4 again). The former effect is consistent with the studies reviewed in the Related Literature Section (see Refs. [42, 43]). The latter effect echoes the findings obtained in several studies on tourism in Thailand. Accordingly, tourists coming to Thailand are sensitive to safety issues in Thailand in general and locally [63–67]. In this context, different studies have focused on different periods. Data from 1981 to 2014 (Harasarn and Chancharat [65], from 2009 to 2016 (Chaivichayachat [66]), and from 2010 to 2017 (Chulaphan and Barahona [67]) have been used and for all periods the result was clear: a lack of any form of safety reduces the demand for tourism in Thailand. Our analysis based on data from 2015 to 2019 corroborates this statement. Quantitatively, the two models differed considerably in the way they captured the effect of the availability of low-cost airlines on tourism receipts. The baseline model states that provinces with low-cost airlines (such as Bangkok, Chonburi, etc., see Table 3) were able to increase their tourism incomes by a factor of 4.7 due to the presence of those low-cost carriers. The fine-grained model suggests an even higher factor of 7.4. This difference across those two factors indicates that the explicit values (4.7 and 7.4) should be interpreted with caution. Nevertheless, the analysis clearly points out that provinces without low-cost airlines such as Samut Prakan and Ayutthaya would profit from having low-cost airlines serving their areas. With respect to the effect of the offence rate, the two models produced qualitatively consistent results (see Table 4: regressions coefficients differed only by a factor 1.01). According to the model Eqs (1) and (2), a decrease of the offence rate by 1 incident per population with 100,000 people increases tourism receipts by 4.8%-6.2% percent (with the higher estimate coming from model 2).

The main effect of occupancy rate showed that during the observation period higher occupancy rates were associated with higher tourism receipts, as expected. As can be read off from Table 4, the models suggest that every 1% increase in occupancy rates increases receipts by 2.5% - 2.9% (with model 2 producing the slightly higher estimate). As mentioned above, while the number of foreign visitors and the occupancy rate are naturally correlate, the two variables exhibited no severe multicollinearity issue. In this context, note that when three tourists arrive in Thailand, they may occupy three rooms (when they are three solo travelers), two rooms (when they are a couple and an individual traveler) or a single room (when they are two parents and a child). For this reason, the number of foreign visitors and hotel occupancy rates are two indicators that capture related but different aspects of tourism demand. Interesting, the two models discussed in the current study favor the two factors differently. While the baseline model produces a more promising gain factor of 4.8% for the number of tourists factor that contrasts with the less promising 3.2% value predicted by the fine-grained model, the fine-grained model favors the occupancy rate factor and predicts a promising gain factor of 2.9% that contrasts with the less enthusiastic value of 2.5% predicted the baseline model.

While the baseline model showed some significant effects for quarters 2 and 3, the fine-grained model showed no statistically significant main effects at all for the factor calendar quarter. At first glance, these results seem to be contradictory. In fact, they may help to better understand the way the factor calendar quarter influenced tourism demand in Thailand during the observation period. Within the two-model framework used in the current study, the baseline model 1 captured the impact of calendar quarter only in terms of the province-unspecific main effect of the factor quarter. In contrast, the fine-grained model 2 took province-specific effects of calendar quarter in terms of the random effect terms (see Table 5) and the province-unspecific effect of calendar quarter in terms of the aforementioned main effect into account. The results presented in Table 4 demonstrate that the province-unspecific main effect disappears when province-specific effects of calendar quarter are taken into account. This, in turn, supports the idea that calendar quarter actually has different impacts for different provinces as reported in Table 5 and that the main effect observed for the baseline model should be interpreted with caution and might be a spurious effect.

**Fig 1:**
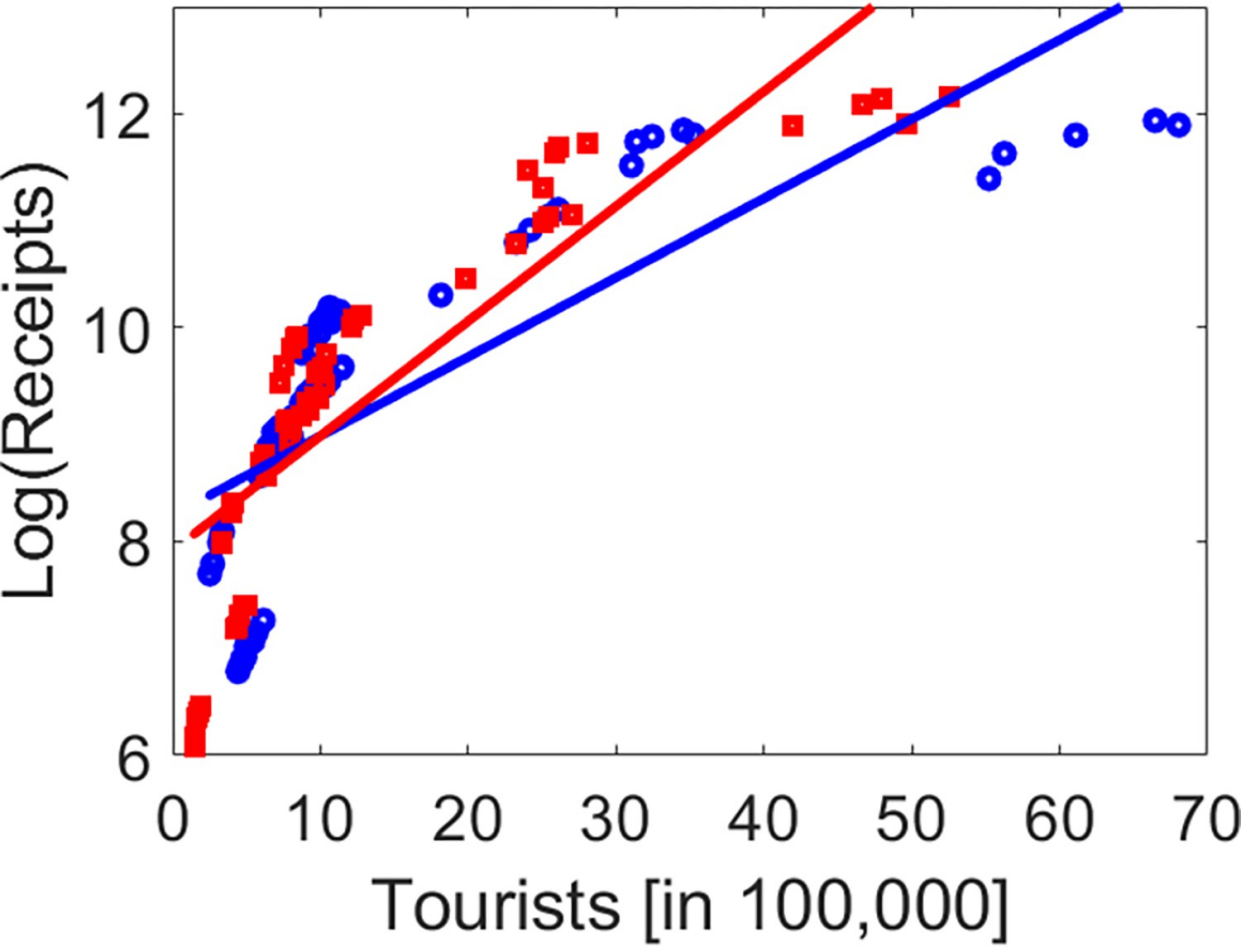
Illustration of the interaction effect between quarter and number of tourists. Log(Receipts) are shown versus number of tourists for Q1 (blue circles) and Q4 (red squares). Linear regression lines are shown as well.

For both models the interaction effect between quarter and the number of foreign visitors was significant and had a positive coefficient for the fourth quarter (see Table 4). Accordingly, when tourist numbers increased during the observation period then receipts increased as well. However, the increase was more dramatic in Q4 as compared to the reference quarter Q1. In this context, Fig 1 shows quartal receipts versus visits for Q1 and Q4. Linear regression lines are shown as well. Clearly, the regression line for Q4 exhibits a steeper slope as compared to the line for Q1, which illustrates the aforementioned interaction effect. As a result, the GLMM models (1) and (2) consistently predict that when tourist numbers increase in Q1 and Q4 by the same amount than the associated increase in receipts is higher in Q4 as compared to Q1, which has certain implications for policy making that will be discussed in the conclusions section.

**Fig 2:**
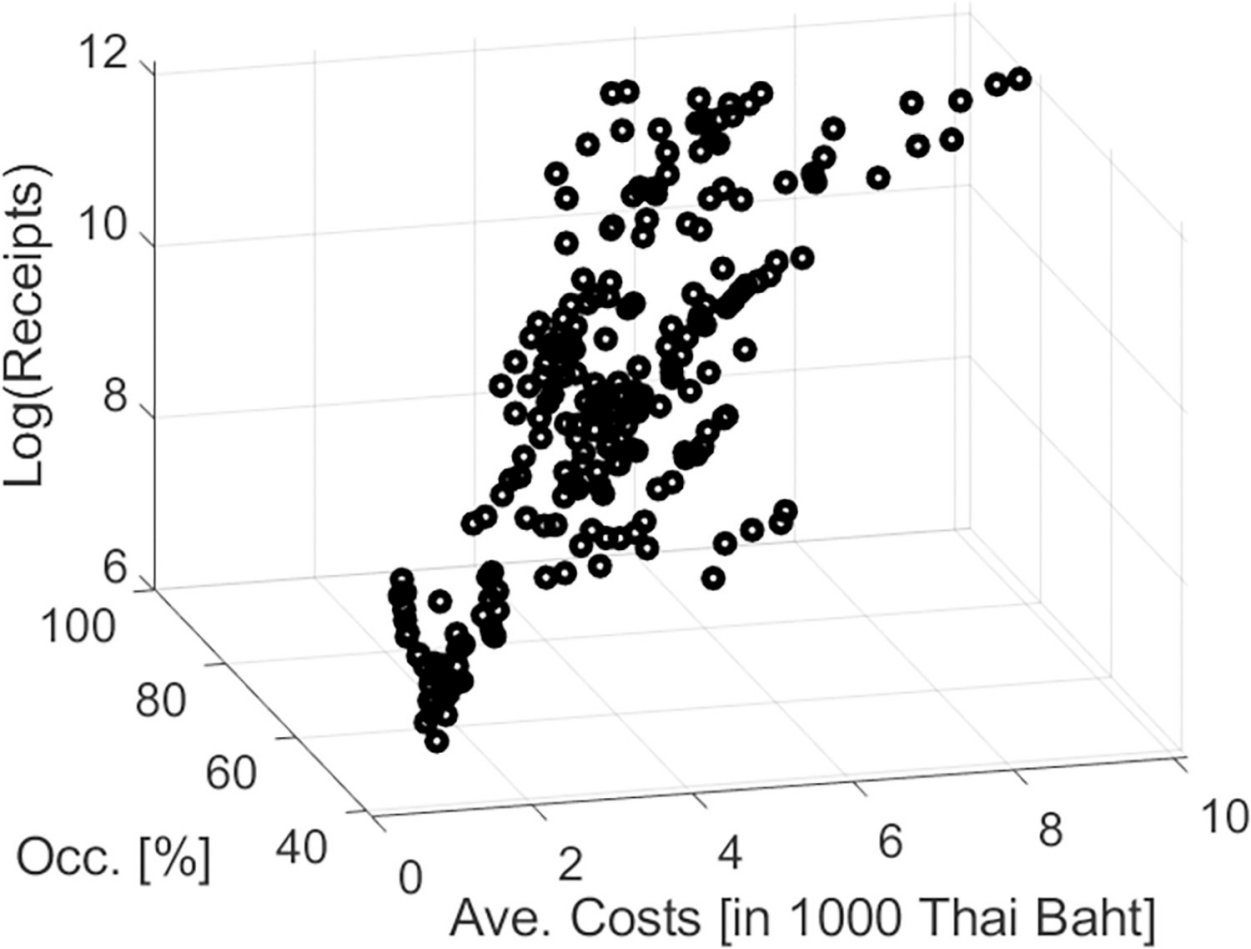
Three-dimensional scatter plot illustrating the interaction between occupancy rate and average cost per day. Log(receipts) are shown versus occupancy rate and average cost per day (abbreviation: occ = occupancy rate). The data cloud forms roughly a banana shaped object that starts at low values in both predictor variables (front bottom corner in the figure) and ends at high values in both predictors (top corner in the back of the figure).

Both models also consistently showed that the interaction between occupancy rate and the average cost per day was statistically significant and negative (see Table 4). Fig 2 shows a scatter plot of the receipts versus occupancy rate and average cost per day. The data cloud forms a shape of a banana that starts at low values for both variables and ends at high values for both variables. In line with the banana-shaped form, the increase of the receipts for low values is relatively high, whereas the increase in income from receipts is relatively low when both variables are close to their maximum values. The negative coefficient of the interaction effect captures this effect. This difference in “gain” for low and high values implies that one should distinguish between two specific markets: the non-saturated budget market, where budget tourists spend small or moderate amounts of money and the occupancy rate is low, and the saturated high-spending market, where occupancy rate is high and involves high-spending tourist. Improving the conditions in terms of increasing either occupancy rate or average cost per day or both would have different effects. Improving the conditions in the first case (non-saturated budget market) would result in a dramatic increase of income from receipts (see Fig 2, the region close to the low values on the x and y axes). In contrast, improving the conditions in the second case would not yield such a dramatic increase (see Fig 2 again, the region with the maximum values for x and y). Consequences of this interaction effect for the parties involved in the tourist industry will be briefly addressed in the conclusions section.

Previous research has found that factors such as cleanness, the exchange rate and related to that price differences between Thailand and other countries and season are factors that influence tourists visiting Thailand [63–67]. In contrast, in the current study air pollution in terms of PM_10_, (which can be considered a proxy for cleanness) and exchange rate as a factor were tested but for both factors in neither of the two models statistically significant effects could be found. In addition, for both models the effect of season was not statistically significant. Having said that, as discussed above, it was found that the factor calendar quarter that can be interpreted as a seasonal variable on a shorter time scale was involved in an interaction effect that determined tourism receipts obtained in Thailand during the observation period 2015–2019. Therefore, this interaction effect may has captured the seasonality effect that was found in the aforementioned studies. Finally, the main effects of the two remaining factors that have not yet been discussed were not statistically significant: confidence index and the number of tourist attractions.

Table 5 shows the estimated fixed and specific random intercepts for models 1 and 2. Let us first turn to model 1. Accordingly, half of the provinces under consideration had statistically significant providence-specific intercepts: Bangkok, Chonburi, Phuket and Songkhla. Conversely, the remaining provinces had intercepts that were not statistically significant: Chiang Mai, Ayutthaya, Samut Prakan and Surat Thani. This implies that the latter provinces share a common model with a particular intercept that holds for all four provinces. In contrast, the provinces of the first group exhibiting province-specific (random) intercepts. In line with the GLMM approach, these intercepts reflect effects on tourism receipt that are not captured by the 12 independent factors considered in model 1. In other words, the analysis suggests that during the observation period there were factors that affected tourism receipt in provinces such as Bangkok, Chonburi, Phuket and Songkhla and caused receipts to differ from the remaining four province. For example, when considering the first two provinces Bangkok and Chiang Mai listed in Table 5, then due these unknown factors receipts from the Bangkok area would be lower by 21% than receipts earned in the Chaing Mai province in the hypothetical situation when both provinces during a certain quarter would have had exactly the same number of tourists, the same offence rate, and so on. That is, Eq (1) predicts that when all factors are the same across the two provinces, then the Chiang Mai province sees a higher tourism income than the Bangkok area. In general, the observation of statistically significant random intercepts (here: in four provinces) should motivate agencies involved in the tourism industry to look for possible, alterative factors not addressed in the current study that could explain these intercepts (we will briefly return to the issue of alternative factors in the Section Conclusions and Limitations). By analogy, similar considerations can be made for the random intercepts of model 2 reported in Table 5. As mentioned above, the random intercept terms of model 2 describes province-specific effects of calendar quarter on tourism receipts. For example, Bangkok province exhibited during the third quarters of the observation period a dramatic effect on receipts as measured in terms of a positive coefficient of 0.6367. Again, according to the GLMM approach, this coefficient captures effects that increased receipts in the Bangkok area during the July-September months of the five years observation period but are not accounted for by the 12 factors included in model 2.

## Conclusions and policy implications

The current study focused on the period 2015–2019 during which Thai tourism reached an all-time high and that has not yet been researched systematically. The study aimed to identify determinants of demand of tourism in Thailand out of a relatively large pool of possible variables and aimed to examine if tourism demand in Thailand is subjected to interaction effects that involve several determinants in combination. A GLMM dual-modeling approach using a baseline model and a fine-grained model was used to address the relevance of unknown factors of tourism demand beyond the aforementioned pool of variables and the relevance of subtle characteristics of tourism receipts data. Five determinants (number of visitors, average cost per day, length of stay, presence of low-cost airlines, and offence rate) were identified and their quantitative impacts on tourism receipts were determined. Moreover, the analysis uncovered two interaction effects (number of visitors with calendar quarter, occupancy rate with average cost per day) and revealed that during the observation period still-to-be-determined, unknown factors indeed played a role for tourism demand in Thailand. The estimation results of our dual-modelling approach also suggest that it is beneficial to test for the possibility of subtle data characteristics as we did in the current study with the help of the fine-grained GLMM approach. Overall, our study is complementary to previous research on demand of tourism in Thailand and sheds some new light on tourism research in general.

When several variables affect tourism demand, as revealed in the current study, then policy makers due to resource limitations may find themselves forced to focus on one variable rather than all of them. In the context of the current study, it was found that a one-hundred thousand increase in the number of tourists produces a 3.2%-4.8% increase in receipts, while increasing the length of stay by 1 day produces a 17%-19% increase in receipts. In view of these numbers, focusing on the length of stay may be more rewarding and may be also more cost-effective than focusing on increasing the number of tourists. In general, when length of stay positively affects spending, then policy makers and tourist agencies should act accordingly [36]. Different approaches may be taken to persuade tourists to stay longer. For example, tourist agencies may offer attractive two-week packages. Specifically for Thailand, during the high season of the year 2022/2023 the Ministry of Interior temporarily extended the 30 days tourist visa to 45 days with the hope that tourists receiving 45 days visa would stay longer than 30 days in Thailand [81]. We suggest that such campaigns may be repeated even on larger scales. The 45-for-30 days campaign may be repeated regularly during the high-season winter months and/or tourist visas for more than 30 days may be offered in general. In fact, a similar suggestion to attract long-term remote workers (“digital nomad”), who work for foreign companies, but live temporarily in Thailand has been made recently by the Krungthai Bank research unit [82].

Studies on determinants of tourism demand in Thailand have pointed out again and again the importance of safety issues. The offence rate in the current study covering the period 2015 to 2019 was a factor that lowered tourism demand. Conversely, assuming that the established models (1) and (2) approximately hold for the post-COVID period, reducing the offence rate just by 1 incident in a reference population of 100,000 people increases tourism demand by 4.8%-6.2%. As reviewed above, due to the COVID-19 pandemic tourists have become even more sensitive to travel risks than they have been before. Therefore, it is plausible to assume that safety issues irrespective of their nature play an even more important role in the ongoing post-COVID era as compared to the pre-COVID period addressed in the current study and that the monetary benefit would actually higher than the estimated 4.8%-6.2% increase. Specifically for Thailand, at this point we can only repeat the conclusion drawn by Chulaphan and Barahona [67] that the Thai government and local tourist operators (e.g., hotels, resorts, entertainment places) should increase their efforts to make Thailand a safe destination. This would not only pay off by boosting tourism but it would increase life quality for the domestic population, that is, for Thai people.

The two interaction effects discovered in the present study can be used to draw two important conclusions. First, from the calendar quarter by number of foreign visitors interaction effect it follows that it is more rewarding to increase tourist arrivals in Q4 as compared to Q1. Consequently, when government agencies and tourist businesses have fixed budgets available to launch campaigns to attract additional tourists, then it pays off better to spend the money to attract Q4 tourists (October to December tourists) rather than Q1 tourists (January to March tourists). Second, in view of the interaction between occupancy rate and average cost per day, we suggest that government tourist agencies and tourism businesses in Thailand should focus more attention to the low-budget market that comes with hotels suffering from a lack of guests. When resources of the relevant government and business agencies are directed to improve the situation in this tourism sector, then the reward in terms of additional income from tourism is likely to be particularly high. In summary, both interaction effects allow to formulate recommendations how to invest resources to maximize the payoff of the invested resources.

This importance of interactions of determinants as demonstrated above for Thailand goes beyond the regional scope of the current study. The present study illustrates that it is important for tourism research on determinants of tourism demand to identify interactions between determinants. Some methodological approaches like the GLMM approach presented above allow to identify interactions. Some approaches (for example, linear time series models) are not suitable for that purpose. Consequently, the recommendation can be made to check in before-hand whether a particular method to identify determinants of tourism demand allows for detecting interactions.

The GLMM approach applied to data from Thailand was able to detect the presence of unknown, yet-to-be-determined factors affecting tourism demand. Explicitly, it was found that for the Bangkok province earnings from tourism would be by 21% lower as compared to the Chiang Mai province if all 12 factors considered in the present study would assume the same levels in both provinces (see Section Results and Discussion). Therefore, the question arises, what are the beneficial factors that make that the Chiang Mai province would perform better? What are the obstacles with which the Bangkok areas seems to struggle and make that it would perform worse? Likewise, in the context of the fine-grained modeling analysis, the question arises, what makes that the Bangkok area in the third quarters of the year would out-beat all other provinces again when assuming that all factors such as number of tourists, and so on, would be constant across provinces. While we do not attempt to answer these questions, at this stage, we would like to point out that the GLMM approach as such allows to identify the presence of such effects. That is, beyond the application to Thailand presented above, the GLMM approach presented above should be considered as a powerful tool in the field of tourism research that allows to obtain insights into the presence of interactions between factors and the presence of unknown factors–on top of the possibility to determine main effects of factors and compare their impacts on a quantitative level.

The present study focused on pre-COVID tourism data, which puts a limitation to the generalizability of the findings reported above. Do they hold for the post-COVID period? On the one hand, as it was stated in the introduction, tourism has seen a steep recovery from the pandemic. Tourism arrivals are worldwide close to the pre-COVID “normal” and for Thailand doubled within just one year. On the other hand, some impacts such as changes in the risk awareness of tourists could turn out to be long-lasting in nature. Irrespective of such considerations, the official WHO-dated post-COVID period started in the third quarter of 2023 and Thailand’s national post-COVID tourism-related period started in the fourth quarter of 2022. Consequently, as of Fall 2023, post-COVID data are scarce. If more post-COVID tourism data is available, it would be a promising endeavor to compare tourism demand between pre- and post-COVID periods. For Thailand, the present study could serve as a reference point in this regard. Returning to the issue of limitations and how they could be overcome in future work, we would like to point out that we used a relatively large set of possible predictor variables. Nevertheless, this set represents a limited selection of possible variables and could be extended. Future work may extend the list of variables used in the present study by other variables frequently used in tourism research such as the GDP and the CPI. In view of several tragic events over the last couple of years, factors such as unexpected war and natural disasters (e.g., floods, wildfires, and earthquakes) may be taken into account as well. In particular, factors should be included that would be promising to explain the statistically significant province-specific random effects reported in Table 5 for model 1 or even the province and quarter specific effects reported in Table 5 for model 2. In the current study, the study area was limited to eight main touristic provinces for which data on PM_10_ was available. Future research on tourism demand in Thailand may include more provinces by either dropping the PM_10_ variable (since its effect was not significant in our study), by measuring air pollution with the help of an alternative and more accessible variable, or by including areas that correspond to minor touristic provinces. If so, spatial effects may be taken into consideration that have been neglected in the current study. For example, between neighboring provinces tourists may engage in 1-day trips (see the detailed description of provinces in the S1 File) which could result in spatial correlations of tourism demand. Such spatial effects have been previously discussed in the literature on tourism demand with the help of more sophisticated methods (see, for example, the study by Ma et al. [83] based on the geographic detector modeling approach).

## Supporting information

S1 File (DOCX)

S1 Fig Histogram of tourism receipts. Empirical histogram of tourism receipts from Thailand during the observation period 2015–2019.
(TIF)

S1 Data Data file. (XLSX)
