# Supplementary material for: Identifying the determinants of tourism receipts of Thailand and relevant determinant-determinant interactions
Source: PLoS One. 2024 Aug 1;19(8):e0308153. doi: 10.1371/journal.pone.0308153 (PMC11293683; doi:10.1371/journal.pone.0308153)
Supplement: S1 File — (DOCX) [file pone.0308153.s001.docx]

**Supporting information**

Analyzing the determinants of tourism receipts of Thailand: a statistical approach allowing to identify determinant-determinant interactions

Khemthong et al.

**SI 1. Descriptions of the provinces constituting the study area**

The provinces are located geographically in different regions of Thailand and exhibit distinct characteristic features [1]. To begin with, Bangkok is the capital of Thailand and is recognized as one of the foremost destinations for tourists. The leisure attractions in Bangkok are historical or religions sites such as temples, palaces, museums, parks, as well as various shopping centers, weekend and night markets. Besides Leisure tourism, Bangkok is also popular for the business tourism including the MICE (Meetings, Incentives, Conferences, and Events), and medical tourism. Samut Prakan is a province located next to the east of Bangkok. Visitors can make day trips from Bangkok to see the leisure attractions in Samut Prakan such as the Ancient City, Erawan museum, and the Crocodile Farm. Interestingly, the Bangkok Suvarnabhumi International Airport is located in Samut Prakan rather than Bangkok province. Phra Nakhon Si Ayutthaya is far from the north of Bangkok just 86 kilometers. It had been the old capital of Thailand that was formerly known as the Kingdom of Ayutthaya. Thailand’s historical and majestic highlights for cultural tourism are storytelling through numerous magnificent temples, palaces, and ruins concentrated in and around the city. Besides their famous temple and historic sites, Ayutthaya's prestigious food are Giant freshwater river prawns and Thai cotton candy. Chon Buri is located on the eastern coast of the Gulf of Thailand, only 80 kilometers from Bangkok. It is Bangkok’s nearest seaside town and is the country’s largest port. The city is a paradise for natural and entertainment attractions with famous beach destinations like Pattaya.

Pattaya is a significant draw for foreign tourists keen to sample its nightlife, golf courses, water sports and diving around Jomtien and Naklua beaches. In addition, Chonburi is a wonderful place for sport tourism such as Scuba diving, and Jet-Ski. Chiang Mai is one of the largest provinces in the northern region of Thailand both in terms of size and economic power. It is a rich city of historical significance. Chiang Mai has its own cultural significance which is distinct from the rest of Thailand. It is the heritage and culture of the community that attract tourists, such as ancient temples, witness vibrant festivals, and indulge in authentic Thai cuisine. Phuket is a province island of Thailand, located in the southern part of the country. It is known for its beautiful beaches, clear water, and vibrant nightlife, such as Rawai, Patong, Karon. Additionally, Phuket is a popular tourist destination due to its rich culture, delicious food, and many historical and natural attractions. Surat Thani is known as “the province of a thousand islands” and located in the southern part of Thailand. The province has the largest area of the entire southern region, most of which is covered by dense forest, and is renowned for its diverse ecosystems, idyllic islands like Ko Samui, Ko Pha-ngan, Ko Tao and Nang Yuan, as well as its golden sands and clear waters. The activities on offer at this province, relating to community tourism, and ecotourism, visitors are also able to learn the local wisdom and see how natural products are made in the community. Songkhla is the province in the south of Thailand that has been nominated as one of cultural heritage site. It is a melting pot of Thais, Chinese and Malays, and charms visitors with its unique traditions, dialect, and folk entertainment. Hat Yai, a district of Songkhla, serves as a hub of communication, trading and transportation as well as a gateway to Malaysia and Singapore, and is the center of economic in the southern region. The old section of Songkhla has maintained its unique identity of ancient and historical flavors through local architecture and cuisine.

**SI 2. Data sources**

Table S1 presents the sources in terms of websites for all variables used in the current study.

**Table S1. Data and their sources**

| Variables | Available at |
| --- | --- |
| Receipts  Foreign visitors  Average cost per day  Calendar season  Calendar quarter  Occupancy rate  Length of stay  Number of tourist attractions  Having low-cost airline  Exchange rate  Air pollution PM10  Crime rate  Size of tourist destination  Tourism confidence index | <https://www.mots.go.th/>.  <https://www.mots.go.th/>.  <https://www.mots.go.th/>.  <https://www.bot.or.th/content/dam/bot/documents/th/research-and-publications/research/discussion-paper-and-policy-paper/250624_WhitepaperVISA.pdf>.  <https://www.mots.go.th/>.  <https://www.mots.go.th/>.  <https://www.mots.go.th/>.  <https://www.traveloka.com/en-th/explore/destination.com>.  <https://www.inthailand.travel/airlines-in-Thailand/#Airlines_in_Thailand_Low_Cost_Airlines>.  <https://www.bot.or.th/en/statistics/exchange-rate.html>.  <http://air4thai.pcd.go.th/webV3/#/History>  <http://thaicrimes.org/crimestat/>  <https://mgronline.com/politics/detail/9620000102759>  <https://www.thailandtourismcouncil.org/product/thailand-tourism-confidence-index>. |

**SI 3. Analysis**

Fig S1 shows the empirical histogram obtained from the data of the entire study area under consideration. The data had a right-skewed distribution. Such distributions have been observed in previous studied (e.g., [2]). The observed skew motivated us to use a Gamma distribution as mentioned above. Table S2 shows the result of the univariate analysis that aimed to determine the predictor variables that should be included in the multivariate model. Accordingly, all variables exhibited p values smaller than 0.25 except for the size of the destination province. In the main text we have taken the argument by Khusnutdinova et al. [3] that was developed for size of cities and assumed that it could be generalized to hold for larger areas such as provinces as well. Our observation that size was ruled out already in the pre-step of our analysis as determinant for tourism demand suggests that such a generalization cannot necessarily be made. Finally, Table S3 shows the variance inflation factors (VIF) of the multicollinearity check for all 12 variables that were accepted in the previous step. All variables showed VIF smaller than 10, which indicates that there were no severe multicollinearity issues among the variables.

**Fig S1. Histogram of tourism receipts**. Empirical histogram of tourism receipts from Thailand during the observation period 2015-2019.

**Table S2. Factors affecting receipts according to our univariate analysis.**

| Factor |  | Estimate | Standard error | P-value |
| --- | --- | --- | --- | --- |
| Calendar season |  | 0.2181 | 0.0440 | < 0.0001 |
| Size of province |  |  |  |  |
| Large |  | 0.9377 | 1.471 | 0.5248 |
| Medium |  | -0.0228 | 1.699 | 0.9893 |
| Small (reference group) |  |  |  |  |
| Calendar quarter |  |  |  |  |
| Q1 (reference group) |  |  |  |  |
| Q2 |  | -0.2145 | 0.0615 | <0.001 |
| Q3 |  | -0.3111 | 0.0614 | <0.0001 |
| Q4 |  | -0.0876 | 0.0613 | 0.1548 |
| Having low-cost airline |  | 3.3976 | 0.7942 | <0.0001 |
| Number of tourist attractions |  | 0.1530 | 0.0574 | <0.01 |
| PM_10_ |  | 0.1539 | 0.0597 | <0.05 |
| Thailand tourism confidence index |  | 0.0814 | 0.0476 | 0.0890 |
| Exchange rate |  | -0.0599 | 0.0128 | < 0.0001 |
| Foreign visitors |  | 0.0464 | 0.0055 | < 0.0001 |
| Average cost per day |  | 0.2951 | 0.0246 | < 0.0001 |
| Average length of stay |  | 0.1502 | 0.0565 | <0.01 |
| Offence rate |  | -0.1316 | 0.0217 | < 0.0001 |
| Occupancy rate |  | 0.0296 | 0.0034 | <0.0001 |

**Table S3. Variance inflation factors of independent variables used in the present study.**

| Variable |  | Variance Inflation |
| --- | --- | --- |
| Calendar season |  | 1.834 |
| Calendar quarter |  | 1.350 |
| Having low-cost airline |  | 3.420 |
| Number of tourist attractions |  | 3.310 |
| PM_10_ |  | 1.792 |
| Thailand tourism confidence index |  | 1.421 |
| Exchange rate |  | 2.273 |
| Foreign visitors |  | 2.642 |
| Average cost per day |  | 3.552 |
| Average length of stay |  | 3.631 |
| Offence rate |  | 2.707 |
| Occupancy rate |  | 4.459 |

**References**

[1] Tourism Authority of Thailand [Internet]. Amazing Thailand – Destinations: 2023 [cited 2023 November 5]. Available from: https://www.tourismthailand.org/Destinations.

[2] Gomez-Deniz E, Perez-Rodríguez JV. Modelling distribution of aggregate expenditure on tourism. Economic Modelling. 2019; 78: 293-308. doi: 10.1016/j.econmod.2018.09.027

[3] Khusnutdinova SR, Sadretdinov DF, Khusnutdinov RR. Tourism as a factor of city development in the postindustrial economy. Advances in Economics. Business and Management Research, 2020; 131: 886–890. doi: 10.2991/aebmr.k.200324.163
