# Supplementary figures and images for: Identifying the determinants of tourism receipts of Thailand and relevant determinant-determinant interactions
Source: PLoS One. 2024 Aug 1;19(8):e0308153. doi: 10.1371/journal.pone.0308153 (PMC11293683; doi:10.1371/journal.pone.0308153)

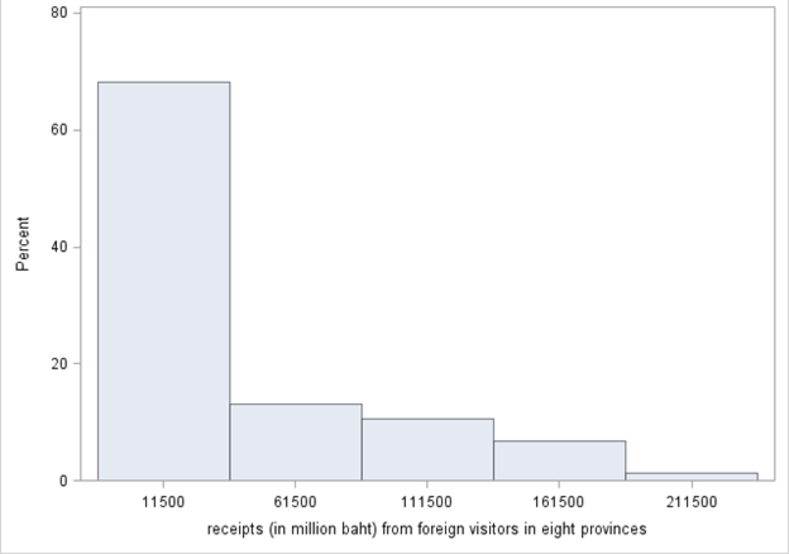

Supplement: S1 Fig — Empirical histogram of tourism receipts from Thailand during the observation period 2015–2019. (TIF) [file pone.0308153.s002.tif]
